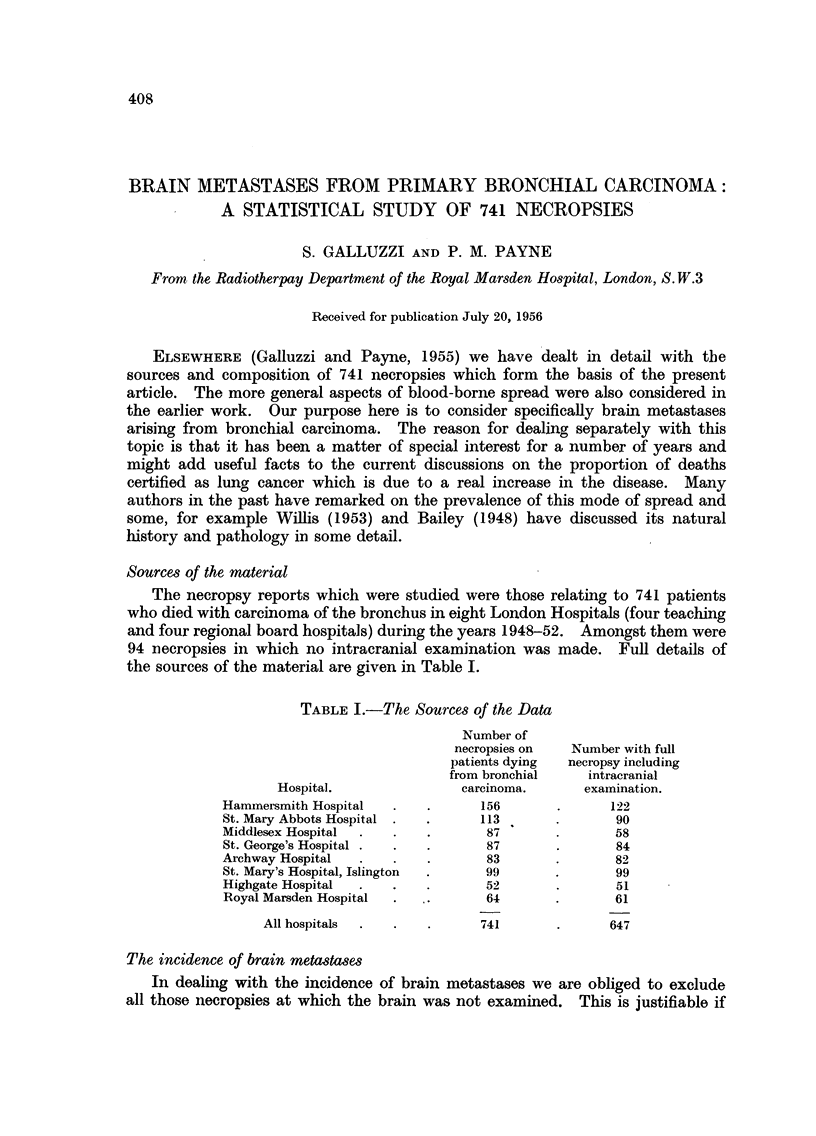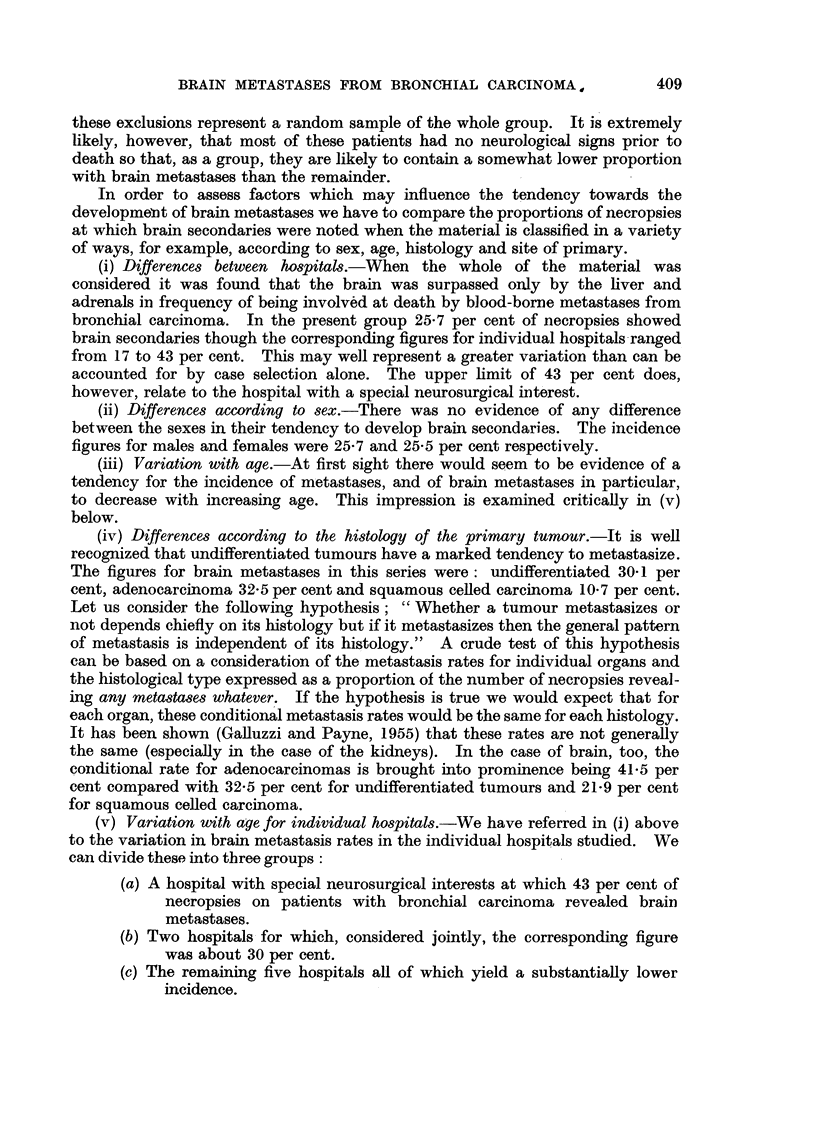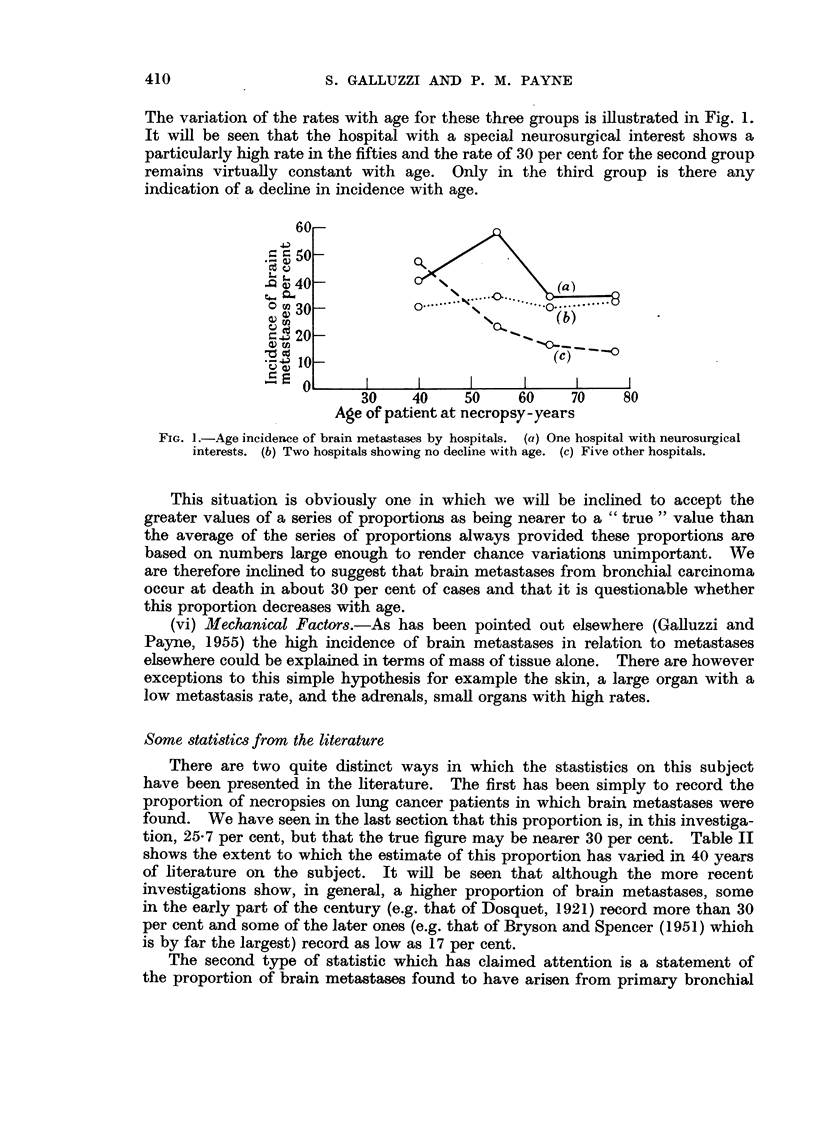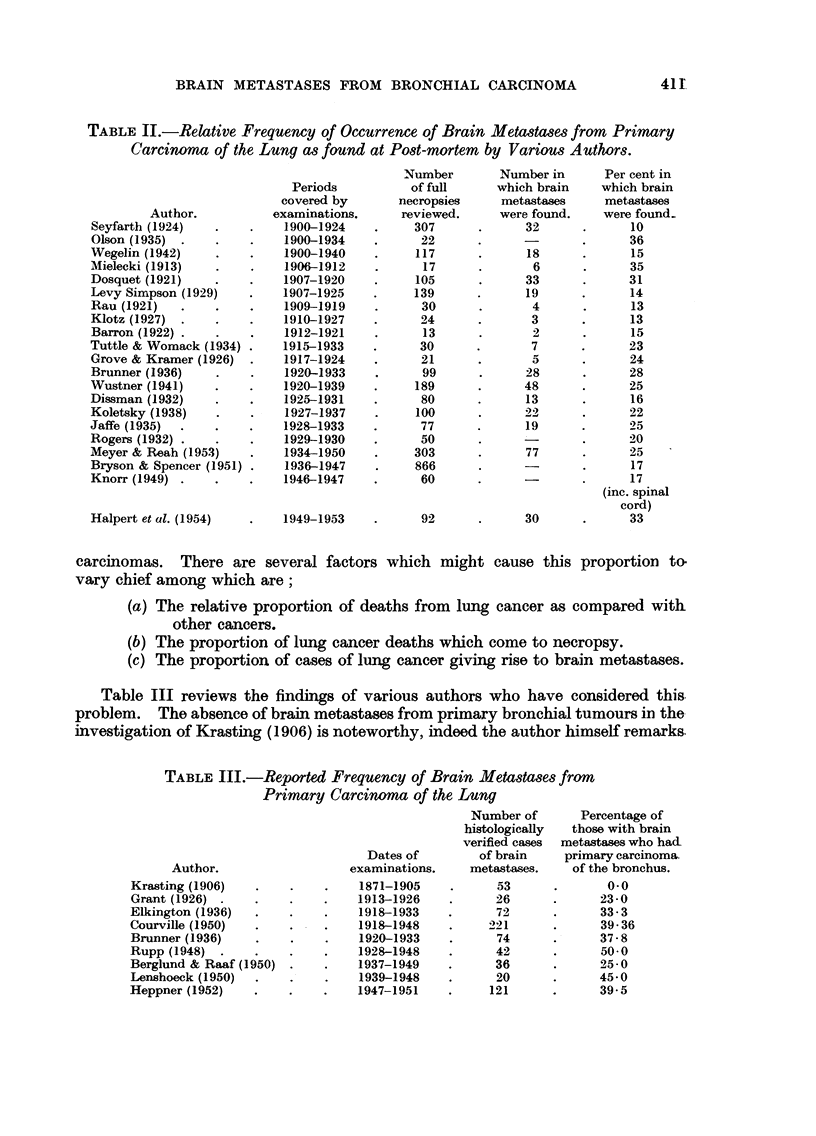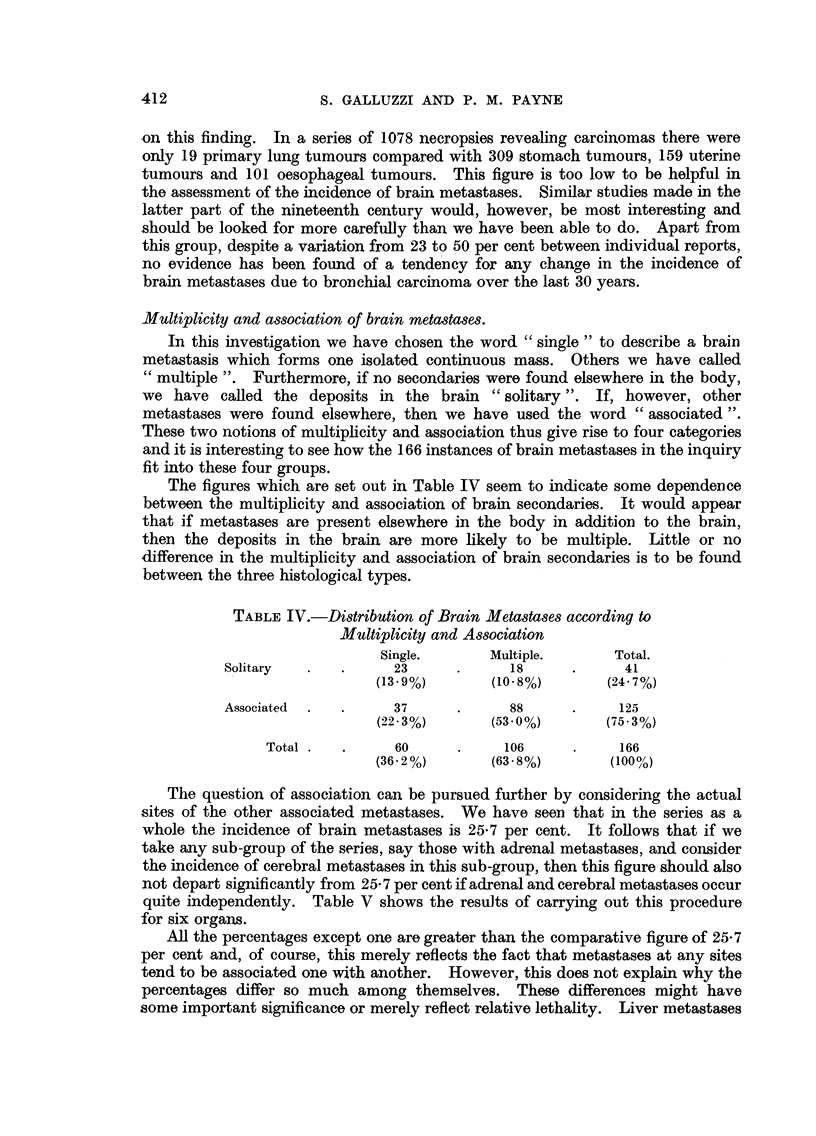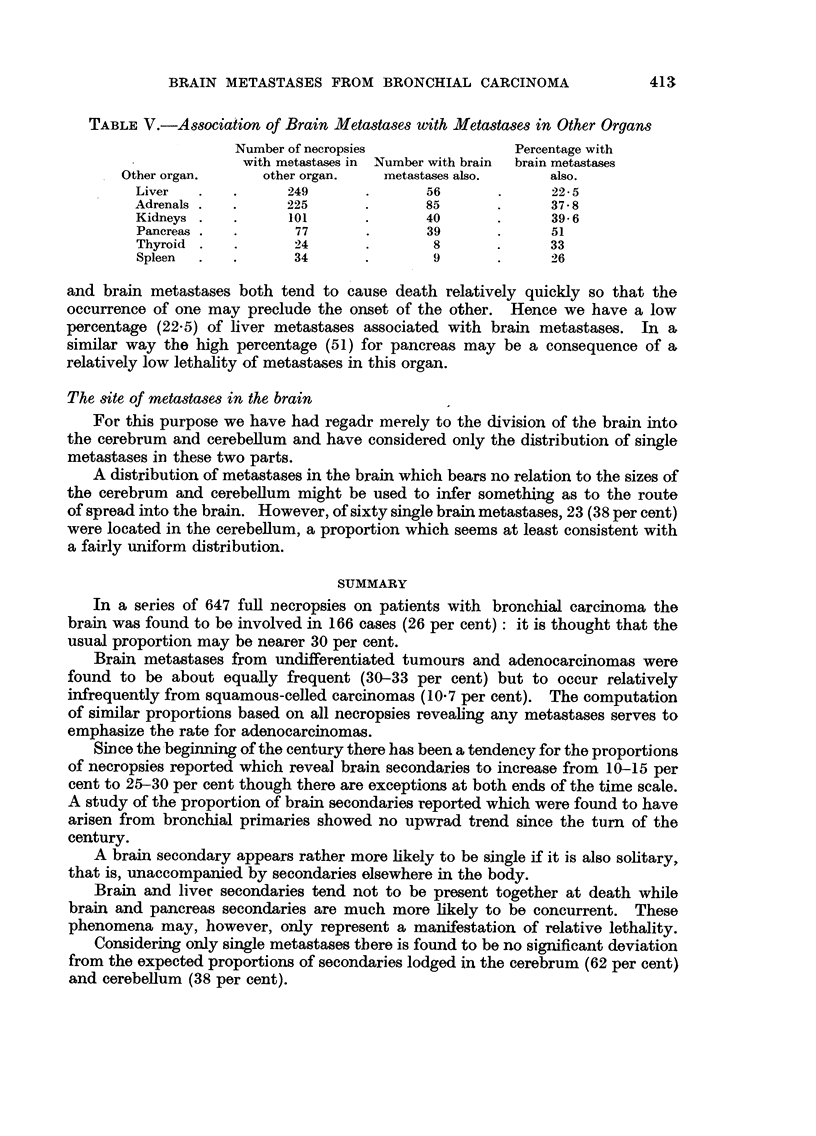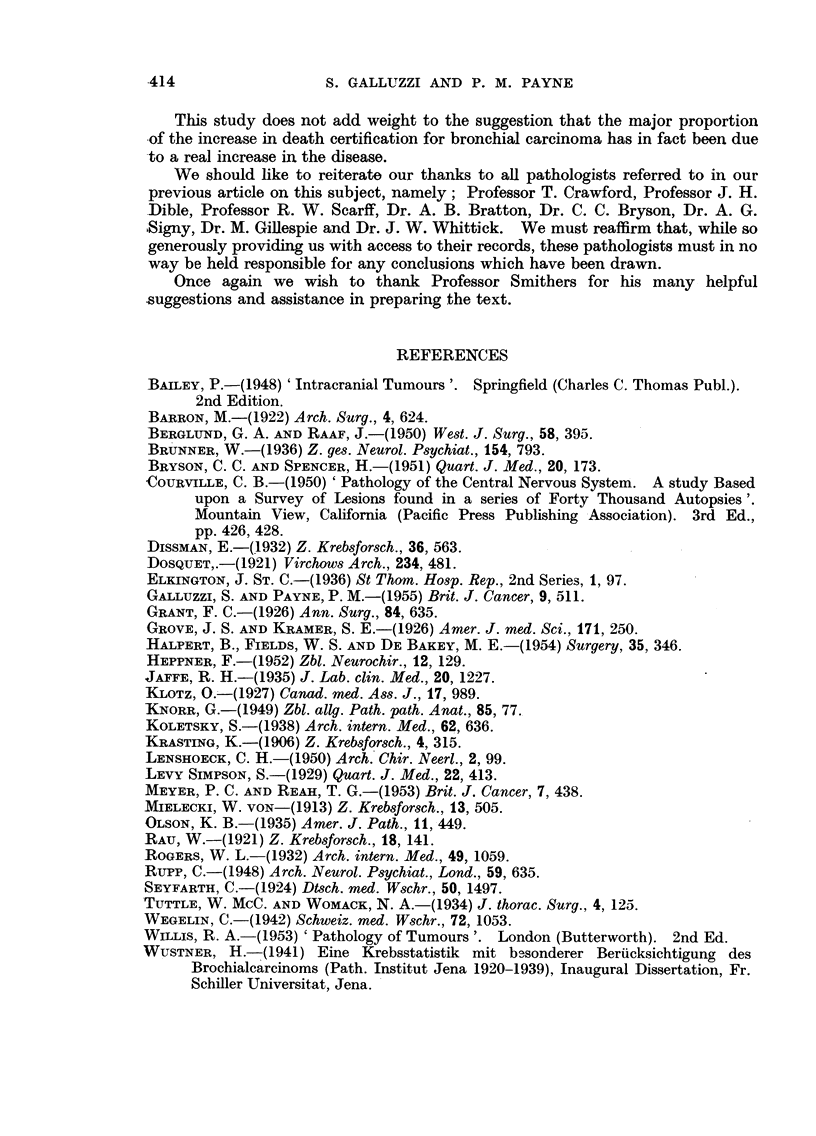# Brain Metastases from Primary Bronchial Carcinoma: A Statistical Study of 741 Necropsies

**DOI:** 10.1038/bjc.1956.47

**Published:** 1956-09

**Authors:** S. Galluzzi, P. M. Payne


### BRAIN METASTASES FROM PRIMARY BRONCHIAL CARCINOMA: A STATISTICAL STUDY OF 741 NECROPSIES

S. GALLUZZI AND P. M. PAYNE

*From the Radiotherpay Department of the Royal Marsden Hospital, London, S.W.3*

Received for publication July 20, 1956

ELSEWHERE (Galluzzi and Payne, 1955) we have dealt in detail with the sources and composition of 741 necropsies which form the basis of the present article. The more general aspects of blood-borne spread were also considered in the earlier work. Our purpose here is to consider specifically brain metastases arising from bronchial carcinoma. The reason for dealing separately with this topic is that it has been a matter of special interest for a number of years and might add useful facts to the current discussions on the proportion of deaths certified as lung cancer which is due to a real increase in the disease. Many authors in the past have remarked on the prevalence of this mode of spread and some, for example Willis (1953) and Bailey (1948) have discussed its natural history and pathology in some detail.

*Sources of the material*

The necropsy reports which were studied were those relating to 741 patients who died with carcinoma of the bronchus in eight London Hospitals (four teaching and four regional board hospitals) during the years 1948–52. Amongst them were 94 necropsies in which no intracranial examination was made. Full details of the sources of the material are given in Table I.

TABLE I.—*The Sources of the Data*

| Hospital. | Number of necropsies on patients dying from bronchial carcinoma. | Number with full necropsy including intracranial examination. |
|---|---|---|
| Hammersmith Hospital | 156 | 122 |
| St. Mary Abbots Hospital | 113 | 90 |
| Middlesex Hospital | 87 | 58 |
| St. George's Hospital | 87 | 84 |
| Archway Hospital | 83 | 82 |
| St. Mary's Hospital, Islington | 99 | 99 |
| Highgate Hospital | 52 | 51 |
| Royal Marsden Hospital | 64 | 61 |
| All hospitals | 741 | 647 |

*The incidence of brain metastases*

In dealing with the incidence of brain metastases we are obliged to exclude all those necropsies at which the brain was not examined. This is justifiable if these exclusions represent a random sample of the whole group. It is extremely likely, however, that most of these patients had no neurological signs prior to death so that, as a group, they are likely to contain a somewhat lower proportion with brain metastases than the remainder.

In order to assess factors which may influence the tendency towards the development of brain metastases we have to compare the proportions of necropsies at which brain secondaries were noted when the material is classified in a variety of ways, for example, according to sex, age, histology and site of primary.

(i) *Differences between hospitals.*—When the whole of the material was considered it was found that the brain was surpassed only by the liver and adrenals in frequency of being involved at death by blood-borne metastases from bronchial carcinoma. In the present group 25·7 per cent of necropsies showed brain secondaries though the corresponding figures for individual hospitals ranged from 17 to 43 per cent. This may well represent a greater variation than can be accounted for by case selection alone. The upper limit of 43 per cent does, however, relate to the hospital with a special neurosurgical interest.

(ii) *Differences according to sex.*—There was no evidence of any difference between the sexes in their tendency to develop brain secondaries. The incidence figures for males and females were 25·7 and 25·5 per cent respectively.

(iii) *Variation with age.*—At first sight there would seem to be evidence of a tendency for the incidence of metastases, and of brain metastases in particular, to decrease with increasing age. This impression is examined critically in (v) below.

(iv) *Differences according to the histology of the primary tumour.*—It is well recognized that undifferentiated tumours have a marked tendency to metastasize. The figures for brain metastases in this series were: undifferentiated 30·1 per cent, adenocarcinoma 32·5 per cent and squamous celled carcinoma 10·7 per cent. Let us consider the following hypothesis; "Whether a tumour metastasizes or not depends chiefly on its histology but if it metastasizes then the general pattern of metastasis is independent of its histology." A crude test of this hypothesis can be based on a consideration of the metastasis rates for individual organs and the histological type expressed as a proportion of the number of necropsies revealing *any metastases whatever.* If the hypothesis is true we would expect that for each organ, these conditional metastasis rates would be the same for each histology. It has been shown (Galluzzi and Payne, 1955) that these rates are not generally the same (especially in the case of the kidneys). In the case of brain, too, the conditional rate for adenocarcinomas is brought into prominence being 41·5 per cent compared with 32·5 per cent for undifferentiated tumours and 21·9 per cent for squamous celled carcinoma.

(v) *Variation with age for individual hospitals.*—We have referred in (i) above to the variation in brain metastasis rates in the individual hospitals studied. We can divide these into three groups:

(*a*) A hospital with special neurosurgical interests at which 43 per cent of necropsies on patients with bronchial carcinoma revealed brain metastases.

(*b*) Two hospitals for which, considered jointly, the corresponding figure was about 30 per cent.

(*c*) The remaining five hospitals all of which yield a substantially lower incidence.

The variation of the rates with age for these three groups is illustrated in Fig. 1. It will be seen that the hospital with a special neurosurgical interest shows a particularly high rate in the fifties and the rate of 30 per cent for the second group remains virtually constant with age. Only in the third group is there any indication of a decline in incidence with age.

FIG. 1.—Age incidence of brain metastases by hospitals. (*a*) One hospital with neurosurgical interests. (*b*) Two hospitals showing no decline with age. (*c*) Five other hospitals.

This situation is obviously one in which we will be inclined to accept the greater values of a series of proportions as being nearer to a " true " value than the average of the series of proportions always provided these proportions are based on numbers large enough to render chance variations unimportant. We are therefore inclined to suggest that brain metastases from bronchial carcinoma occur at death in about 30 per cent of cases and that it is questionable whether this proportion decreases with age.

(vi) *Mechanical Factors.*—As has been pointed out elsewhere (Galluzzi and Payne, 1955) the high incidence of brain metastases in relation to metastases elsewhere could be explained in terms of mass of tissue alone. There are however exceptions to this simple hypothesis for example the skin, a large organ with a low metastasis rate, and the adrenals, small organs with high rates.

*Some statistics from the literature*

There are two quite distinct ways in which the stastistics on this subject have been presented in the literature. The first has been simply to record the proportion of necropsies on lung cancer patients in which brain metastases were found. We have seen in the last section that this proportion is, in this investigation, 25·7 per cent, but that the true figure may be nearer 30 per cent. Table II shows the extent to which the estimate of this proportion has varied in 40 years of literature on the subject. It will be seen that although the more recent investigations show, in general, a higher proportion of brain metastases, some in the early part of the century (e.g. that of Dosquet, 1921) record more than 30 per cent and some of the later ones (e.g. that of Bryson and Spencer (1951) which is by far the largest) record as low as 17 per cent.

The second type of statistic which has claimed attention is a statement of the proportion of brain metastases found to have arisen from primary bronchial

TABLE II.—*Relative Frequency of Occurrence of Brain Metastases from Primary Carcinoma of the Lung as found at Post-mortem by Various Authors.*

| Author. | Periods covered by examinations. | Number of full necropsies reviewed. | Number in which brain metastases were found. | Per cent in which brain metastases were found. |
|---|---|---|---|---|
| Seyfarth (1924) | 1900–1924 | 307 | 32 | 10 |
| Olson (1935) | 1900–1934 | 22 | — | 36 |
| Wegelin (1942) | 1900–1940 | 117 | 18 | 15 |
| Mielecki (1913) | 1906–1912 | 17 | 6 | 35 |
| Dosquet (1921) | 1907–1920 | 105 | 33 | 31 |
| Levy Simpson (1929) | 1907–1925 | 139 | 19 | 14 |
| Rau (1921) | 1909–1919 | 30 | 4 | 13 |
| Klotz (1927) | 1910–1927 | 24 | 3 | 13 |
| Barron (1922) | 1912–1921 | 13 | 2 | 15 |
| Tuttle & Womack (1934) | 1915–1933 | 30 | 7 | 23 |
| Grove & Kramer (1926) | 1917–1924 | 21 | 5 | 24 |
| Brunner (1936) | 1920–1933 | 99 | 28 | 28 |
| Wustner (1941) | 1920–1939 | 189 | 48 | 25 |
| Dissman (1932) | 1925–1931 | 80 | 13 | 16 |
| Koletsky (1938) | 1927–1937 | 100 | 22 | 22 |
| Jaffe (1935) | 1928–1933 | 77 | 19 | 25 |
| Rogers (1932) | 1929–1930 | 50 | — | 20 |
| Meyer & Reah (1953) | 1934–1950 | 303 | 77 | 25 |
| Bryson & Spencer (1951) | 1936–1947 | 866 | — | 17 |
| Knorr (1949) | 1946–1947 | 60 | — | 17 (inc. spinal cord) |
| Halpert *et al.* (1954) | 1949–1953 | 92 | 30 | 33 |

carcinomas. There are several factors which might cause this proportion to vary chief among which are ;

(*a*) The relative proportion of deaths from lung cancer as compared with other cancers.
(*b*) The proportion of lung cancer deaths which come to necropsy.
(*c*) The proportion of cases of lung cancer giving rise to brain metastases.

Table III reviews the findings of various authors who have considered this problem. The absence of brain metastases from primary bronchial tumours in the investigation of Krasting (1906) is noteworthy, indeed the author himself remarks

TABLE III.—*Reported Frequency of Brain Metastases from Primary Carcinoma of the Lung*

| Author. | Dates of examinations. | Number of histologically verified cases of brain metastases. | Percentage of those with brain metastases who had primary carcinoma of the bronchus. |
|---|---|---|---|
| Krasting (1906) | 1871–1905 | 53 | 0·0 |
| Grant (1926) | 1913–1926 | 26 | 23·0 |
| Elkington (1936) | 1918–1933 | 72 | 33·3 |
| Courville (1950) | 1918–1948 | 221 | 39·36 |
| Brunner (1936) | 1920–1933 | 74 | 37·8 |
| Rupp (1948) | 1928–1948 | 42 | 50·0 |
| Berglund & Raaf (1950) | 1937–1949 | 36 | 25·0 |
| Lenshoeck (1950) | 1939–1948 | 20 | 45·0 |
| Heppner (1952) | 1947–1951 | 121 | 39·5 |

on this finding. In a series of 1078 necropsies revealing carcinomas there were only 19 primary lung tumours compared with 309 stomach tumours, 159 uterine tumours and 101 oesophageal tumours. This figure is too low to be helpful in the assessment of the incidence of brain metastases. Similar studies made in the latter part of the nineteenth century would, however, be most interesting and should be looked for more carefully than we have been able to do. Apart from this group, despite a variation from 23 to 50 per cent between individual reports, no evidence has been found of a tendency for any change in the incidence of brain metastases due to bronchial carcinoma over the last 30 years.

*Multiplicity and association of brain metastases.*

In this investigation we have chosen the word " single " to describe a brain metastasis which forms one isolated continuous mass. Others we have called " multiple ". Furthermore, if no secondaries were found elsewhere in the body, we have called the deposits in the brain " solitary ". If, however, other metastases were found elsewhere, then we have used the word " associated ". These two notions of multiplicity and association thus give rise to four categories and it is interesting to see how the 166 instances of brain metastases in the inquiry fit into these four groups.

The figures which are set out in Table IV seem to indicate some dependence between the multiplicity and association of brain secondaries. It would appear that if metastases are present elsewhere in the body in addition to the brain, then the deposits in the brain are more likely to be multiple. Little or no difference in the multiplicity and association of brain secondaries is to be found between the three histological types.

TABLE IV.—*Distribution of Brain Metastases according to Multiplicity and Association*

| | Single. | Multiple. | Total. |
|---|---|---|---|
| Solitary | 23<br>(13·9%) | 18<br>(10·8%) | 41<br>(24·7%) |
| Associated | 37<br>(22·3%) | 88<br>(53·0%) | 125<br>(75·3%) |
| Total | 60<br>(36·2%) | 106<br>(63·8%) | 166<br>(100%) |

The question of association can be pursued further by considering the actual sites of the other associated metastases. We have seen that in the series as a whole the incidence of brain metastases is 25·7 per cent. It follows that if we take any sub-group of the series, say those with adrenal metastases, and consider the incidence of cerebral metastases in this sub-group, then this figure should also not depart significantly from 25·7 per cent if adrenal and cerebral metastases occur quite independently. Table V shows the results of carrying out this procedure for six organs.

All the percentages except one are greater than the comparative figure of 25·7 per cent and, of course, this merely reflects the fact that metastases at any sites tend to be associated one with another. However, this does not explain why the percentages differ so much among themselves. These differences might have some important significance or merely reflect relative lethality. Liver metastases

TABLE V.—*Association of Brain Metastases with Metastases in Other Organs*

| Other organ. | Number of necropsies with metastases in other organ. | Number with brain metastases also. | Percentage with brain metastases also. |
|---|---|---|---|
| Liver | 249 | 56 | 22·5 |
| Adrenals | 225 | 85 | 37·8 |
| Kidneys | 101 | 40 | 39·6 |
| Pancreas | 77 | 39 | 51 |
| Thyroid | 24 | 8 | 33 |
| Spleen | 34 | 9 | 26 |

and brain metastases both tend to cause death relatively quickly so that the occurrence of one may preclude the onset of the other. Hence we have a low percentage (22·5) of liver metastases associated with brain metastases. In a similar way the high percentage (51) for pancreas may be a consequence of a relatively low lethality of metastases in this organ.

*The site of metastases in the brain*

For this purpose we have had regadr merely to the division of the brain into the cerebrum and cerebellum and have considered only the distribution of single metastases in these two parts.

A distribution of metastases in the brain which bears no relation to the sizes of the cerebrum and cerebellum might be used to infer something as to the route of spread into the brain. However, of sixty single brain metastases, 23 (38 per cent) were located in the cerebellum, a proportion which seems at least consistent with a fairly uniform distribution.

### SUMMARY

In a series of 647 full necropsies on patients with bronchial carcinoma the brain was found to be involved in 166 cases (26 per cent) : it is thought that the usual proportion may be nearer 30 per cent.

Brain metastases from undifferentiated tumours and adenocarcinomas were found to be about equally frequent (30–33 per cent) but to occur relatively infrequently from squamous-celled carcinomas (10·7 per cent). The computation of similar proportions based on all necropsies revealing any metastases serves to emphasize the rate for adenocarcinomas.

Since the beginning of the century there has been a tendency for the proportions of necropsies reported which reveal brain secondaries to increase from 10–15 per cent to 25–30 per cent though there are exceptions at both ends of the time scale. A study of the proportion of brain secondaries reported which were found to have arisen from bronchial primaries showed no upwrad trend since the turn of the century.

A brain secondary appears rather more likely to be single if it is also solitary, that is, unaccompanied by secondaries elsewhere in the body.

Brain and liver secondaries tend not to be present together at death while brain and pancreas secondaries are much more likely to be concurrent. These phenomena may, however, only represent a manifestation of relative lethality.

Considering only single metastases there is found to be no significant deviation from the expected proportions of secondaries lodged in the cerebrum (62 per cent) and cerebellum (38 per cent).

This study does not add weight to the suggestion that the major proportion of the increase in death certification for bronchial carcinoma has in fact been due to a real increase in the disease.

We should like to reiterate our thanks to all pathologists referred to in our previous article on this subject, namely ; Professor T. Crawford, Professor J. H. Dible, Professor R. W. Scarff, Dr. A. B. Bratton, Dr. C. C. Bryson, Dr. A. G. Signy, Dr. M. Gillespie and Dr. J. W. Whittick. We must reaffirm that, while so generously providing us with access to their records, these pathologists must in no way be held responsible for any conclusions which have been drawn.

Once again we wish to thank Professor Smithers for his many helpful suggestions and assistance in preparing the text.

### REFERENCES

BAILEY, P.—(1948) ' Intracranial Tumours '. Springfield (Charles C. Thomas Publ.). 2nd Edition.
BARRON, M.—(1922) *Arch. Surg.*, **4**, 624.
BERGLUND, G. A. AND RAAF, J.—(1950) *West. J. Surg.*, **58**, 395.
BRUNNER, W.—(1936) *Z. ges. Neurol. Psychiat.*, **154**, 793.
BRYSON, C. C. AND SPENCER, H.—(1951) *Quart. J. Med.*, **20**, 173.
COURVILLE, C. B.—(1950) ' Pathology of the Central Nervous System. A study Based upon a Survey of Lesions found in a series of Forty Thousand Autopsies '. Mountain View, California (Pacific Press Publishing Association). 3rd Ed., pp. 426, 428.
DISSMAN, E.—(1932) *Z. Krebsforsch.*, **36**, 563.
DOSQUET,.—(1921) *Virchows Arch.*, **234**, 481.
ELKINGTON, J. ST. C.—(1936) *St Thom. Hosp. Rep.*, 2nd Series, **1**, 97.
GALLUZZI, S. AND PAYNE, P. M.—(1955) *Brit. J. Cancer*, **9**, 511.
GRANT, F. C.—(1926) *Ann. Surg.*, **84**, 635.
GROVE, J. S. AND KRAMER, S. E.—(1926) *Amer. J. med. Sci.*, **171**, 250.
HALPERT, B., FIELDS, W. S. AND DE BAKEY, M. E.—(1954) *Surgery*, **35**, 346.
HEPPNER, F.—(1952) *Zbl. Neurochir.*, **12**, 129.
JAFFE, R. H.—(1935) *J. Lab. clin. Med.*, **20**, 1227.
KLOTZ, O.—(1927) *Canad. med. Ass. J.*, **17**, 989.
KNORR, G.—(1949) *Zbl. allg. Path. path. Anat.*, **85**, 77.
KOLETSKY, S.—(1938) *Arch. intern. Med.*, **62**, 636.
KRASTING, K.—(1906) *Z. Krebsforsch.*, **4**, 315.
LENSHOECK, C. H.—(1950) *Arch. Chir. Neerl.*, **2**, 99.
LEVY SIMPSON, S.—(1929) *Quart. J. Med.*, **22**, 413.
MEYER, P. C. AND REAH, T. G.—(1953) *Brit. J. Cancer*, **7**, 438.
MIELECKI, W. VON—(1913) *Z. Krebsforsch.*, **13**, 505.
OLSON, K. B.—(1935) *Amer. J. Path.*, **11**, 449.
RAU, W.—(1921) *Z. Krebsforsch.*, **18**, 141.
ROGERS, W. L.—(1932) *Arch. intern. Med.*, **49**, 1059.
RUPP, C.—(1948) *Arch. Neurol. Psychiat., Lond.*, **59**, 635.
SEYFARTH, C.—(1924) *Dtsch. med. Wschr.*, **50**, 1497.
TUTTLE, W. McC. AND WOMACK, N. A.—(1934) *J. thorac. Surg.*, **4**, 125.
WEGELIN, C.—(1942) *Schweiz. med. Wschr.*, **72**, 1053.
WILLIS, R. A.—(1953) ' Pathology of Tumours '. London (Butterworth). 2nd Ed.
WUSTNER, H.—(1941) Eine Krebsstatistik mit besonderer Berücksichtigung des Brochialcarcinoms (Path. Institut Jena 1920–1939), Inaugural Dissertation, Fr. Schiller Universitat, Jena.